# Pre-operative Serum Albumin as a Predictor of Adverse Outcomes in Open Abdominal Surgery: A Retrospective Study in Central Queensland

**DOI:** 10.7759/cureus.79681

**Published:** 2025-02-26

**Authors:** Sophia Bee Ting Tan, Xiaohui Lin, Muhammad Farqhan Rosley, Michael Lamparelli

**Affiliations:** 1 General Surgery, Rockhampton Hospital, Rockhampton, AUS

**Keywords:** abdominal laparotomy wounds, albumin level, morbidity and mortality, primary laparotomy, wound infections

## Abstract

Purpose

Hypoalbuminemia, which is indicative of protein-energy malnutrition, is linked to increased morbidity and mortality in acute surgical patients due to increased catabolism. This study evaluates the relationship between preoperative serum albumin levels and postoperative morbidity and mortality in patients who underwent open abdominal surgery.

Methodology

We used the Operating Room Management Information System (ORMIS) and coding to retrospectively find a complete list of surgeries from January 2021 to June 2023 using the term “laparotomy” and “open surgery”. Patient demographics, comorbidities, pre-operative serum albumin, laboratory parameters, surgical category, postoperative complications, length of hospital stay, and death up to postoperative day 30 were collected. Serum albumin less than 35g/L is considered as hypoalbuminemia in this study.

Results

A total of 182 patients were analyzed, with 101 having hypoalbuminemia (serum albumin<35 g/L) and 81 having normal albumin levels (serum albumin≥35 g/L). Patients with hypoalbuminemia had significantly lower haemoglobin levels (126 g/L vs. 142 g/L, p<0.001) and higher American Society of Anaesthesiologists (ASA) scores, with more patients classified as ASA 4 and 5 (33.7% and 12.9% vs. 14.8% and 9.9%, p=0.024). The median hospital stay was longer in the hypoalbuminemia group (10 vs. 7 days, p=0.006). Wound dehiscence was more frequent (11.9% vs. 2.5%, p=0.018), and mortality was significantly higher (11.9% vs. 3.7%, p=0.046), highlighting the association between low preoperative albumin and worse surgical outcomes.

Conclusions

Preoperative serum albumin effectively predicts surgical outcomes after open abdominal surgery. It serves as a valuable, cost-effective risk predictor that should be further explored for integration into preoperative assessments.

## Introduction

Preoperative risk assessment plays a crucial role in predicting postoperative outcomes and guiding perioperative management. The American Society of Anaesthesiologists (ASA) classification, combined with the urgency and complexity of the surgery, has been shown to correlate with postoperative morbidity and mortality [1,2].

While several preoperative assessment protocols exist to evaluate the risk of complications, nutritional status remains a critical but often underutilized factor in surgical risk prediction. Malnutrition is frequently observed in surgical patients and is associated with higher rates of complications and prolonged hospital stays [3,4]. Effective preoperative nutritional screening can help identify patients who may benefit from targeted interventions to optimize their surgical outcomes. However, the lack of universally accepted diagnostic criteria for malnutrition presents a challenge [5].

Various clinical and laboratory parameters are utilized for nutritional assessment, with the European Society for Clinical Nutrition and Metabolism (ESPEN) recommending a combination of unintentional weight loss and either a low BMI or a reduced fat-free mass index (FFMI) [6]. The concepts of cachexia and sarcopenia highlight the significance of changes in fat and muscle mass as predictors of clinical outcomes. Compared to basic metrics like body mass (BM), the two-compartment model: fat mass index (FMI) and FFMI, provides a more accurate evaluation. Since FFMI is a key determinant, advanced body composition measurement techniques are increasingly necessary. However, devices such as bioelectrical impedance analyzers and DXA scanners are not routinely accessible in clinical practice.

Several serum markers have been explored for assessing malnutrition, including serum proteins such as albumin and prealbumin, as well as retinol-binding protein (RBP), transferrin, total cholesterol, and inflammatory markers like C-reactive protein (CRP) and total lymphocyte count (TLC) [7]. Serum albumin, the most abundant plasma protein, is essential for maintaining oncotic pressure, transporting vital substances, and reflecting the body's nutritional and inflammatory status [8]. It has long been used as an adjunct for risk stratification in preoperative evaluations [9]. Hypoalbuminemia, defined as serum albumin levels below 35 g/L, has been strongly associated with adverse surgical outcomes, including impaired wound healing, higher infection rates, prolonged hospitalization, and increased mortality [10]. Despite its potential, the routine use of serum albumin in preoperative risk assessment remains controversial, as it is influenced by underlying disease states rather than nutritional status alone [8]. While factors such as hydration status and inflammation can influence albumin levels, it remains one of the most commonly utilized markers due to its ease of measurement, affordability, reproducibility, and strong predictive value for surgical outcomes [7]. Compared to other serum markers, albumin consistently responds to nutritional interventions, making it a practical and reliable tool in clinical practice.

This retrospective study aims to evaluate the predictive value of preoperative serum albumin levels in patients undergoing open abdominal surgery in Central Queensland, Australia. By analyzing its association with postoperative complications, mortality, and length of hospital stay, this study seeks to determine whether serum albumin should be integrated into routine preoperative risk assessment protocols to enhance surgical outcomes.

## Materials and methods

This retrospective analysis included patients who underwent open abdominal surgery between January 2021 and June 2023 in Rockhampton Hospital, Queensland, Australia. Ethical review for this study was exempted by the Central Queensland Hospital and Health Service Human Research Ethics Committee (HREC) (EX/2025/QCQ/115764) and has been endorsed for publication.

Study design and patient selection

This retrospective cohort study was conducted at Rockhampton Hospital, Queensland, Australia, from January 2021 to June 2023. The study analyzed patients who underwent open abdominal surgery during this period. Surgical cases were identified through the Operating Room Management Information System (ORMIS) and hospital coding data using the search terms “laparotomy” and “open” to capture all relevant cases.

A total of 217 patients were initially identified. To ensure consistency in the dataset, only the first surgical procedure within the study period was considered for each patient. Exclusion criteria were applied to remove cases that did not align with the study objectives, resulting in the exclusion of 35 patients for the following reasons: non-primary operations (n=11); missing preoperative serum albumin levels or levels measured more than 30 days prior to surgery (n=8); interhospital transfers (n=11); surgeries involving other non-general surgical specialties (n=3); patients under the age of 18 years (n=1); and surgeries performed with palliative intent (n=1)

**Figure 1:**
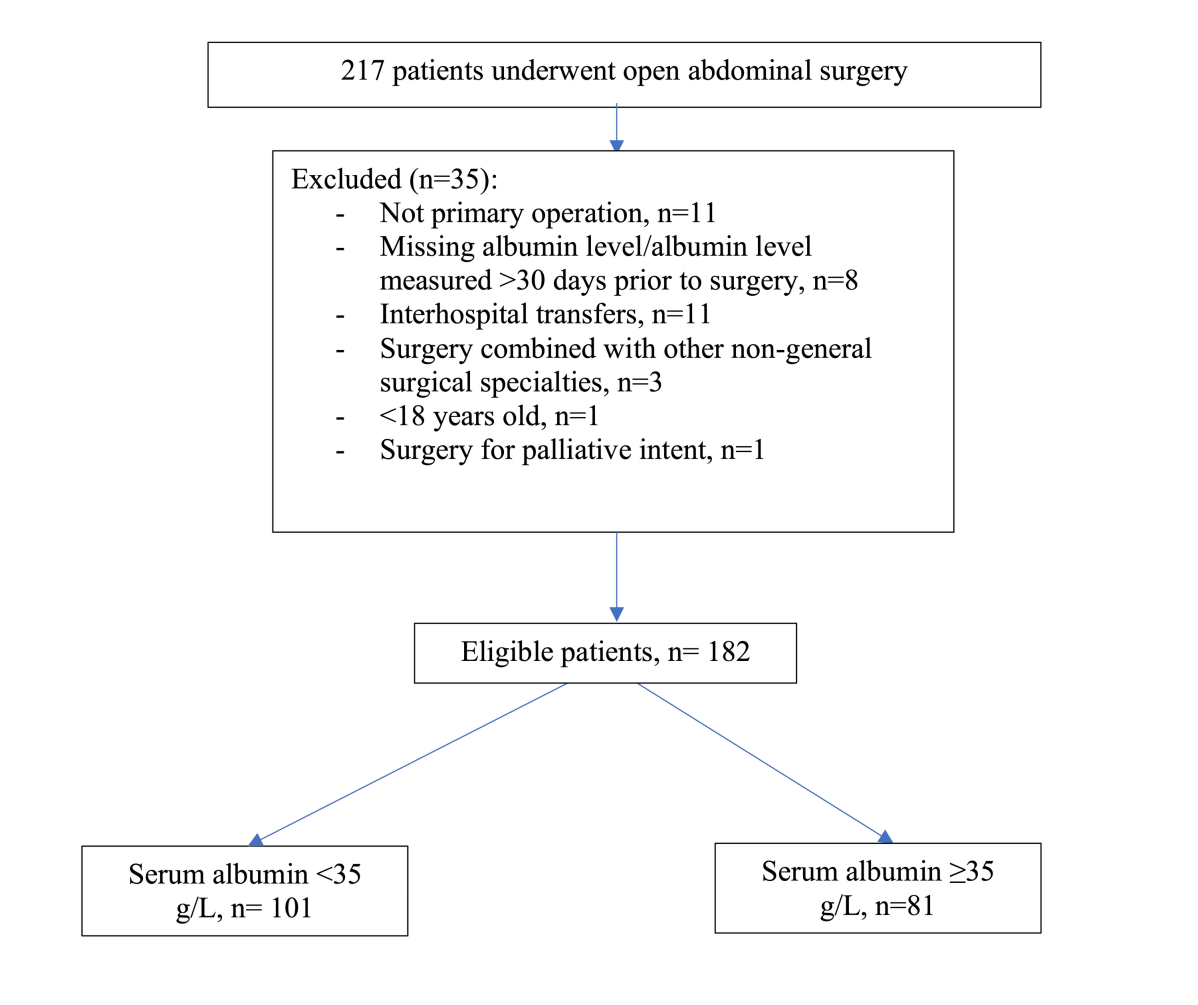
Patient selection flow chart.

After applying these exclusions, 182 patients met the eligibility criteria and were included in the final analysis (Figure 1).

Data collection and study variables

Data was extracted from the electronic medical records and included demographics, comorbid conditions, ASA classification, surgical setting (emergency vs. elective), and preoperative laboratory parameters. The preoperative laboratory parameters analyzed included serum albumin levels, haemoglobin, and white cell count.

Serum albumin levels were specifically assessed as a marker of nutritional status, with hypoalbuminemia defined as a preoperative serum albumin level<35 g/L. Surgical procedures were categorized into six primary groups based on anatomical location: gastroduodenal, biliary, small bowel, appendix, colorectal, and miscellaneous procedures (including herniotomy, adhesiolysis, splenectomy, stoma revision/reversal, and diagnostic procedures). Cases involving procedures spanning multiple categories were classified separately.

Outcome measures

The primary aim of the study was to assess the relationship between preoperative serum albumin levels and postoperative outcomes. The key postoperative outcomes assessed included: wound-related complications, intra-abdominal collections, anastomotic leaks, in-hospital mortality, and length of hospital stay.

Statistical analysis

Statistical analysis was conducted using SPSS Statistics 30.0 (IBM Corp, version 30.0, Armonk, NY, USA). Continuous variables were summarized using means and standard deviations and compared using a student’s t-test. Categorical variables were expressed as frequencies and percentages and analyzed using the Chi-square test or Fisher’s exact test, as appropriate. A two-tailed p-value<0.05 was considered statistically significant. Additional subgroup analyses were performed to explore the association between hypoalbuminemia and specific postoperative complications.

All statistical tests were reviewed for appropriateness, and missing data were handled using listwise deletion to maintain the integrity of the dataset. Potential confounders, including patient comorbidities, surgical urgency, and perioperative interventions, were considered in the analysis to enhance the validity of the findings.

## Results

A total of 182 patients who underwent open abdominal surgery were included in the study, with 101 patients (55.5%) having preoperative serum albumin levels below 35 g/L and 81 patients (44.5%) having levels≥35 g/L. The median age of patients in the hypoalbuminemia group (serum albumin<35 g/L) was 67 years (IQR: 57-77), compared to 63 years (IQR: 48-73) in the normal albumin group (p=0.096), indicating no statistically significant difference. The gender distribution was similar between the two groups, with 57 (56.4%) males and 44 (43.6%) females in the hypoalbuminemia group, and 43 (53.1%) males and 38 (46.9%) females in the normal albumin group (p=0.652). A significant difference was observed in ASA classification between the two groups (p=0.024). A higher proportion of patients in the hypoalbuminemia group had ASA scores of 4 (33.7% vs. 14.8%) and 5 (12.9% vs. 9.9%), while the normal albumin group had a higher proportion of patients with ASA scores of 3 (51.9% vs. 36.6%) and 1 (6.2% vs. 2%). No significant difference was found in the surgical setting, with emergency surgeries comprising 94.1% and 93.8% in the hypoalbuminemia and normal albumin groups, respectively (p=1.000). Comorbid conditions did not show statistically significant differences between the groups. However, haemoglobin levels were significantly lower in the hypoalbuminemia group (126 g/L, IQR: 111-143) compared to the normal albumin group (142 g/L, IQR: 132-153), with a p-value<0.001, indicating statistical significance. White cell count levels were comparable between the groups (p=0.240) (Table 1).

**Table 1:** Patient baseline characteristics. The data is presented as median (interquartile range, IQR) for continuous variables and frequency (percentage) for categorical variables. Statistical significance was set at p<0.05. Student’s t-test was used to compare continuous variables (Age, Hb, and WCC) between the two groups. Chi-square test or Fisher’s exact test was used for categorical variables (gender, ASA classification, setting, and comorbidities). Hb; haemoglobin; WCC: white cell count.

Baseline characteristics	Serum albumin <35 g/L, n= 101	Serum albumin >= 35 g/L, n=81	Test-statistical value	p-value
Age (years), median (IQR)	67 (57-77)	63 (48-73)	1.674	0.096
Gender, n (%)			0.204	0.652
Male	57 (56.4)	43 (53.1)		
Female	44 (43.6)	38 (46.9)		
ASA classification, n (%)			11.287	0.024
1	2 (2)	5 (6.2)		
2	14 (14.9)	14 (17.3)		
3	37 (36.6)	42 (51.9)		
4	34 (33.7)	12 (14.8)		
5	13 (12.9)	8 (9.9)		
Setting, n (%)			-	1.000
Emergency	95 (94.1)	76 (93.8)		
Elective	6 (5.9)	5 (6.2)		
Co-morbidities, n (%)				
Diabetes	15 (14.9)	7 (8.6)	1.631	0.202
Chronic kidney disease	12 (11.9)	6 (7.4)	1.010	0.315
Chronic liver disease	2 (2)	0	-	0.503
Chronic heart failure/Ischaemic heart disease	18 (17.8)	8 (9.9)	2.317	0.128
Chronic obstructive pulmonary disease	15 (14.9)	10 (12.3)	0.238	0.626
Malignancy	22 (21.8)	9 (11.1)	3.622	0.057
Hypertension	36 (35.6)	30 (37)	0.038	0.846
Dyslipidaemia	20 (19.8)	18 (22.2)	0.159	0.690
Laboratory parameters, median (IQR)				
Hb (g/L)	126 (111-143)	142 (132-153)	-4.932	<0.001
WCC (x 10^9^/L)	11.6 (8.3 – 16.2)	11.7 (9-15.3)	1.180	0.240

The most common surgical categories were herniotomy, adhesiolysis, splenectomy, stoma revision/reversal, and diagnostic procedures, accounting for 42.3% of the cases. Colorectal procedures were the second most frequent at 31.9%, followed by small bowel surgery at 15.4%. Other categories include gastroduodenal (6%), appendix (3.3%), biliary (0.5%), and multiple procedures (0.5%) (Table 2).

**Table 2:** Distribution of surgical categories among study patients. The data is presented as frequency (percentage) for each surgical category. Surgical procedures were classified into six primary categories (gastroduodenal, biliary, small bowel, appendix, colorectal, and miscellaneous procedures including herniotomy, adhesiolysis, splenectomy, stoma revision/reversal, and diagnostic procedures). Cases involving procedures spanning multiple categories were classified separately.

	Surgical category, n=182	n (%)
1.	Gastroduodenal	11 (6)
2.	Biliary	1 (0.5)
3.	Small bowel	28 (15.4)
4.	Appendix	6 (3.3)
5.	Colorectal	58 (31.9)
6.	Herniotomy, adhesiolysis, splenectomy, stoma revision/reversal and diagnostic purpose	77 (42.3)
7.	More than one category	1 (0.5)

The median length of hospital stay was significantly longer in patients with hypoalbuminemia at 10 days (IQR: 7-15), compared to 7 days (IQR: 4-10) in those with normal albumin levels (p=0.006). Postoperative morbidity was higher in the hypoalbuminemia group, with wound dehiscence occurring in 11.9% of patients compared to 2.5% in the normal albumin group, a statistically significant difference (p=0.018). Other complications, including surgical site infection (8.9% vs. 4.9%, p=0.301), intra-abdominal collection (8% vs. 4.9%, p=0.410), and anastomotic leak (4% vs. 1.2%, p=0.383), were more frequent in the hypoalbuminemia group, although these differences were not statistically significant. Mortality was significantly higher in the hypoalbuminemia group, with a rate of 11.9% compared to 3.7% in the normal albumin group (p=0.046), indicating a statistically significant association between hypoalbuminemia and increased mortality risk (Table 3).

**Table 3:** Association of preoperative serum albumin levels with postoperative outcomes. The data is presented as median (interquartile range, IQR) for continuous variables and frequency (percentage) for categorical variables. Statistical significance was set at p<0.05. Student’s t-test was used to compare continuous variables (Length of Stay). Chi-square test or Fisher’s exact test was used for categorical variables (morbidity and mortality).

Outcomes	Serum albumin <35 g/L, n=101	Serum albumin >= 35 g/L, n=81	Test-statistical value	p-value
Length of stay (days), median (IQR)	10 (7-15)	7 (4-10)	2.764	0.006
Morbidity, n (%)				
Wound dehiscence	12 (11.9)	2 (2.5)	5.608	0.018
Surgical site infection	9 (8.9)	4 (4.9)	1.070	0.301
Intra-abdominal collection	8 (8)	4 (4.9)	0.649	0.420
Anastomotic leak	4 (4)	1 (1.2)	-	0.383
Mortality, n (%)	12 (11.9)	3 (3.7)	3.975	0.046

## Discussion

This study demonstrates that preoperative hypoalbuminemia (serum albumin<35 g/L) is significantly associated with adverse postoperative outcomes in patients undergoing open abdominal surgery. Patients with low serum albumin levels experienced prolonged hospital stays (10 vs. 7 days, p=0.006), higher rates of wound dehiscence (11.9% vs. 2.5%, p=0.018), and increased mortality (11.9% vs. 3.7%, p=0.046) compared to those with normal albumin levels. Furthermore, hypoalbuminemia was strongly correlated with higher ASA classifications (p=0.024), reflecting a greater overall surgical risk. These findings reinforce the importance of preoperative serum albumin as a valuable prognostic marker in surgical risk stratification and perioperative planning.

Serum albumin, the most abundant plasma protein, plays a critical role in maintaining colloid osmotic pressure and transporting essential molecules [11]. Its synthesis is primarily regulated by colloid osmotic pressure and dietary protein intake, with a plasma half-life of 15-19 days, making it an important indicator of the body's metabolic and physiological state [12]. Hypoalbuminemia is commonly observed in hospitalized and critically ill patients and is associated with unfavorable outcomes. Inflammatory cytokines such as tumour necrosis factor-alpha (TNF-α) and Interleukin-6 (IL-6), released during physiological stressors like infection, surgery, or trauma, contribute to decreased serum albumin levels by increasing vascular permeability, enhancing protein degradation, and reducing hepatic synthesis [11,13]. This highlights the complex interplay between inflammation and serum albumin levels in the perioperative period.

Serum albumin is widely recognized as a biochemical and nutritional marker in the preoperative assessment of patients undergoing various surgical procedures, including cardiac, trauma, and general surgeries [14,15]. Several studies have demonstrated its prognostic value. In a cohort of 400 patients undergoing cardiac surgery, hypoalbuminemia was linked to prolonged intensive care unit (ICU) stays and extended hospitalization [16]. Similarly, in colorectal cancer surgery, low preoperative serum albumin levels were associated with an increased risk of surgical complications such as surgical site infections and prolonged hospital stays (OR 1.79, p<0.05) [17]. A prospective observational study on emergency exploratory laparotomies further emphasized the association between hypoalbuminemia and adverse postoperative outcomes, including wound dehiscence and prolonged hospitalization [15]. A large cohort study conducted in Denmark, which included 3,639 patients over the age of 60 who underwent open abdominal surgery, found that non-survivors had significantly lower mean serum albumin levels compared to survivors (20.6 g/L vs. 30.1 g/L, p<0.0001) [12]. A study conducted in Thailand identified a dose-response relationship between serum albumin levels and the risk of in-hospital mortality among patients undergoing gastrointestinal surgery, demonstrating that patients with severe hypoalbuminemia (<2.0 mg/dL) had a 2.0-2.5 times higher risk of mortality compared to those with non-severe hypoalbuminemia (≥2.0-3.4 g/dL) [17]. 

It is essential to acknowledge that albumin serves as a negative acute-phase protein, with its circulating levels influenced by a range of inflammatory conditions and pharmacologic agents, particularly those impacting hepatic function. Clinical states such as hepatic failure, major burns, sepsis, trauma, postoperative recovery, and malignancy have been well-documented to suppress serum albumin concentrations [18]. Consequently, the presence of hypoalbuminemia should be interpreted within the broader context of the patient’s pathophysiological status rather than being exclusively attributed to malnutrition.

One of the major challenges in diagnosing malnutrition is the absence of a standardized definition. Various assessment tools are utilized to evaluate malnutrition, primarily relying on laboratory markers (serum-based indicators) and physical examination findings. Among these, the Subjective Global Assessment (SGA) is the most widely implemented tool. It includes a detailed medical history encompassing weight loss, alterations in dietary intake, gastrointestinal symptoms, and functional status, combined with a physical examination evaluating subcutaneous fat depletion, muscle wasting, and the presence of edema or ascites. Recent studies have validated SGA as an effective method for nutritional assessment in both medical and surgical inpatients, suggesting its potential superiority over other screening tools in the early identification of malnutrition [19].

However, some evidence suggests that serum albumin measurement may still serve as a valuable diagnostic tool for assessing malnutrition, particularly in patients undergoing cardiac transplantation and orthopedic procedures. A study involving 60 cardiac transplant recipients, evaluated at least five years post-transplant, demonstrated that serum albumin was a more reliable predictor of malnutrition compared to body mass index (BMI) and the SGA [20]. Similarly, in elective orthopedic surgery, preoperative serum albumin levels have been utilized to identify and optimize at-risk patients, contributing to the reduction of postoperative complications in these patients [21].

Conventional clinical assessment methods, such as BMI and weight loss, may inadequately estimate surgical risk, particularly in regions with a high prevalence of obesity, such as the United States [22]. In patients with obesity, significant preoperative cachexia may go undetected using standard evaluation tools, despite profound metabolic and anthropometric alterations. Furthermore, while serum albumin is frequently used as a biomarker of nutritional status, its reliability as an indicator of malnutrition in non-inflammatory conditions, such as starvation, remains questionable [18,7]. A meta-analysis of 63 studies, encompassing 2,125 patients, investigated the impact of prolonged starvation on serum albumin levels in otherwise healthy individuals. The findings indicated that albumin concentrations remained within the normal range until patients reached severe states of malnutrition (BMI < 12 or starvation duration exceeding six weeks), at which point clinical manifestations of malnutrition were already apparent [23]. These findings highlight the limitations of conventional assessment methods and serum albumin in detecting early malnutrition, particularly in non-inflammatory conditions and obese populations, underscoring the need for more sensitive and comprehensive nutritional evaluation tools. 

Therefore, integrating inflammatory markers and validated nutritional screening tools is essential for improving perioperative risk stratification and optimizing patient management.

Limitations

This study has several limitations, primarily due to its retrospective design, which relies on existing electronic medical records and may introduce biases from incomplete or missing data. The exclusion of patients with missing values could lead to selection bias, potentially affecting the representativeness of the study population and influencing the observed associations. Additionally, the reliance on a single preoperative serum albumin measurement may not fully capture the patient’s nutritional status, as it can be influenced by factors such as hydration, inflammation, and liver function. The study's setting in a single geographic region (Central Queensland) may limit the generalizability of the findings to broader populations. Furthermore, the classification of surgical procedures may not account for individual case complexity, and the study primarily focuses on short-term postoperative outcomes, overlooking long-term recovery and quality-of-life measures. Lastly, as an observational study, it cannot establish causality between serum albumin levels and surgical outcomes, necessitating prospective research to validate its findings.

## Conclusions

This study demonstrates that preoperative hypoalbuminemia is significantly associated with adverse postoperative outcomes, including prolonged hospital stays, increased wound-related complications, and higher mortality in patients undergoing open abdominal surgery. These findings support the use of serum albumin as a cost-effective prognostic marker for preoperative risk assessment and perioperative optimization. However, its interpretation must consider the influence of underlying inflammatory and disease processes, as albumin is a negative acute-phase reactant. Additionally, its reliability as a nutritional marker is limited in both acute inflammatory conditions and non-inflammatory states such as prolonged starvation. Relying solely on serum albumin for nutritional assessment may lead to misclassification, and underestimating malnutrition in certain patient populations. Future research should focus on incorporating serum albumin into comprehensive preoperative assessment models while integrating additional validated nutritional screening tools to enhance the accuracy of surgical risk prediction and guide personalized perioperative management strategies.
